# Association of nutritional–immune indicators and their dynamic changes with disease severity and wound healing in diabetic foot ulcers treated by tibial transverse transport: a retrospective study

**DOI:** 10.3389/fnut.2026.1863436

**Published:** 2026-10-08

**Authors:** Yueyue Zhu, Sijie Yang, Zhenhong Zhu, Ruiqing Mo, Ruibin Feng, Xingyu Chen, Dapeng Yu, Hongjie Su, Qikai Hua

**Affiliations:** 1 Collaborative Innovation Centre of Regenerative Medicine and Medical BioResource Development and Application Co-constructed by the Province and Ministry, Guangxi Medical University, Nanning, Guangxi, China; 2 Department of Bone and Joint Surgery, The People’s Hospital of Guangxi Zhuang Autonomous Region, Guangxi Academy of Medical Sciences, Nanning, Guangxi, China; 3 Department of Burn and Plastic Surgery, Children’s Hospital of Soochow University, Suzhou, Jiangsu, China; 4 Department of Diabetic Foot Limb-Salvage and Ulcer Reconstruction, The First Affiliated Hospital of Guangxi Medical University, Nanning, Guangxi, China

**Keywords:** diabetic foot ulcer, nutrition, prognosis, prognostic nutritional index, tibial transverse transport

## Abstract

**Background:**

Although tibial transverse transport (TTT) may promote wound repair in complex diabetic foot ulcer (DFU), reliable indicators reflecting postoperative recovery remain limited. This study aimed to investigate the association of nutritional–immune indicators and their dynamic changes with DFU severity and wound healing time after TTT treatment.

**Methods:**

This retrospective cohort study included 227 DFU patients who underwent TTT between January 2023 and May 2024. Patients were classified according to Wagner grades. LASSO regression and multivariable logistic regression were used to identify nutritional–immune factors associated with DFU severity, with ROC curve analysis, calibration analysis, and decision curve analysis performed to evaluate model performance. In addition, 147 patients with complete 1-month postoperative laboratory data were analyzed using Cox proportional hazards regression, Kaplan–Meier analysis and time-dependent ROC analysis to evaluate the association between dynamic nutritional–immune changes and subsequent wound healing time.

**Results:**

Compared with non-severe DFU patients, severe DFU patients showed greater inflammatory activation and nutritional–immune disturbances. Multivariable logistic regression identified ANLR (OR = 0.993, 95% CI: 0.987–0.999, *p* = 0.020) and PNI (OR = 0.909, 95% CI: 0.853–0.969, *p* = 0.004) as independently associated with DFU severity. The model incorporating ANLR and PNI showed good discrimination for severe DFU (AUC = 0.825, 95% CI: 0.756–0.893). Baseline PNI and ANLR were not associated with postoperative wound healing time, whereas greater ΔPNI was independently associated with a higher hazard of subsequent wound healing in the severity-stratified Cox model (HR = 1.030, 95% CI: 1.008–1.053, *p* = 0.008). Kaplan–Meier analysis showed faster wound healing in patients with greater ΔPNI (log-rank *p* = 0.018).

**Conclusion:**

PNI and ANLR are associated with DFU severity and may provide complementary information for preoperative risk stratification. An increase in PNI at 1 month after TTT is associated with subsequent wound healing time and may represent an integrated response of nutritional–immune status during the treatment course.

## 1 Introduction

Diabetic foot ulcer is one of the most severe complications of diabetes mellitus, with an estimated lifetime incidence of approximately 19–34% among individuals with diabetes (1). DFU is associated with high rates of recurrence, lower-extremity amputation, and mortality, imposing substantial clinical and socioeconomic burdens worldwide (2). The development and progression of DFU represent a complex pathological process involving multiple interacting factors, including peripheral arterial disease (PAD), diabetic peripheral neuropathy (DPN), infection, impaired tissue repair, and systemic metabolic abnormalities (3). In addition to local tissue damage, systemic comorbidities such as renal dysfunction and heart failure and glycemic control have also been reported to influence clinical outcomes in patients with DFU (4). Therefore, comprehensive evaluation of both local and systemic factors is essential for improving risk stratification and management of DFU.

For patients with DFU who fail to respond to conventional treatments, TTT has emerged as an effective limb-salvage strategy and has demonstrated promising outcomes in promoting wound healing (5, 6). Previous studies have suggested that the potential mechanisms of TTT may involve enhancement of microcirculation, promotion of angiogenesis, and modulation of inflammatory responses within the wound microenvironment (7). However, the biological mechanisms underlying the therapeutic effects of TTT remain incompletely understood, and considerable heterogeneity exists in postoperative wound healing trajectories among patients. Some individuals achieve rapid wound closure, whereas others require prolonged treatment and repeated wound management. Previous studies have identified local factors, such as foot temperature and transcutaneous oxygen pressure, as potential indicators associated with poor healing after TTT (8). However, these parameters mainly reflect local vascular or tissue oxygenation status and may not fully capture the influence of systemic metabolic, nutritional, and immune conditions on wound recovery.

Nutritional and immune status are increasingly recognized as important determinants of disease progression and clinical outcomes, acting as an important link between inflammation, metabolism, and host defense mechanisms (9, 10). Patients with diabetes may develop chronic low-grade systemic inflammation even before the onset of DFU, while persistent wound exposure and infection can further aggravate inflammatory responses and disrupt nutritional and immune homeostasis (11, 12). Conventional biomarkers, such as serum albumin, C-reactive protein (CRP), and neutrophil-to-lymphocyte ratio (NLR), reflect only individual aspects of nutritional, inflammatory, or immune status and may not adequately represent the overall biological condition of the host. In contrast, composite nutritional–immune indicators, including the prognostic nutritional index (PNI) and albumin-to-neutrophil-to-lymphocyte ratio (ANLR), integrate information related to nutritional reserves, systemic inflammation, and immune function, thereby providing a more comprehensive assessment of host immune-metabolic balance (12, 13). In the context of DFU, where ischemia, infection, metabolic dysfunction, and impaired tissue repair coexist, systemic nutritional–immune status may not only contribute to disease severity but also influence subsequent wound healing processes and limb outcomes (14).

Although several studies have investigated the prognostic factors after TTT, most previous studies have focused primarily on preoperative inflammatory or immune-related indicators. For example, elevated NLR, monocyte-to-neutrophil ratio (MNR), and platelet-to-lymphocyte ratio (PLR) have been associated with prolonged wound healing time after TTT (15, 16). However, these inflammatory markers mainly represent a single dimension of systemic response and do not fully reflect the interaction between nutritional status, immune function, and inflammation. Moreover, whether dynamic changes in nutritional–immune indicators during the course of TTT are associated with subsequent wound healing remains unclear.

Therefore, this study aimed to comprehensively evaluate nutritional–immune indicators and their dynamic changes in patients with DFU undergoing TTT. Specifically, we investigated the association between nutritional–immune status and DFU severity and further explored whether perioperative changes in these indicators were associated with subsequent wound healing.

## 2 Materials and methods

### 2.1 Study population

This single-center retrospective cohort study consecutively enrolled patients with diabetic foot ulcers (DFUs) who underwent tibial transverse transport (TTT) at the First Affiliated Hospital of Guangxi Medical University between January 2023 and May 2024. The study protocol was approved by the institutional ethics committee, and the requirement for informed consent was waived because of the retrospective design, minimal risk to participants, and use of de-identified clinical data.

A total of 387 consecutive patients undergoing TTT were screened. Patients were eligible if they met the diagnostic criteria for diabetes mellitus according to the American Diabetes Association (ADA), were aged ≥18 years, and fulfilled the indications for TTT. Eligible patients had at least one ulcer located below the ankle joint that persisted for ≥6 months or failed to heal after conventional treatments, including debridement, revascularization, skin substitutes, skin grafting, or local/free flap transplantation. The exclusion criteria were as follows: (1) severe peripheral artery disease (PAD) requiring vascular intervention, defined as the presence of rest pain or persistent limb pain accompanied by >50% arterial lumen stenosis and/or monophasic Doppler waveform, with vascular occlusion caused by atherosclerosis or calcification, with or without an ankle–brachial index (ABI) < 0.5; (2) severe systemic infection or extra-wound infection, including systemic inflammatory response syndrome (SIRS) or sepsis; (3) severe dysfunction of major organs (heart, lung, liver, or brain), or a history of stroke, myocardial infarction, or respiratory distress syndrome within the previous 3 months; (4) renal failure requiring renal replacement therapy or estimated glomerular filtration rate (eGFR) < 30 mL/min/1.73m^2^; (5) Ongoing treatment with glucocorticoids, immunosuppressive agents, or chemotherapy; (6) previous amputation or repeated TTT procedures; (7) incomplete clinical data or unavailable wound healing follow-up information.

### 2.2 Surgical procedure and postoperative management

All patients underwent comprehensive imaging and laboratory evaluations before surgery. Albumin supplementation was administered when serum albumin levels were <30 g/L, and blood transfusion was considered when hemoglobin levels were <70 g/L. TTT procedures were performed according to our previously reported protocol (17). Briefly, patients were placed in the supine position without tourniquet application and underwent spinal anesthesia or femoral nerve block anesthesia. Two longitudinal incisions (approximately 1 cm each) were made at the anteromedial proximal tibia to expose the periosteum. A cortical bone block measuring approximately 5 × 1.5 cm was created using a mini oscillating saw. A 3-mm Kirschner wire was inserted through the bone block, and two 5-mm Schanz screws were placed on each side across both cortices. An external distraction frame was subsequently assembled, and intraoperative fluoroscopy was used to confirm the position of fixation devices and integrity of the bone block. Aggressive wound debridement was performed according to the Infectious Diseases Society of America (IDSA) guidelines, including removal of infected bone tissue when necessary. Patients with wound infection received targeted antibiotic therapy based on microbiological culture and antimicrobial susceptibility testing. Postoperative antibiotic treatment was administered according to clinical assessment and infection status. Daily wound dressing changes and pin-site care were performed. Radiographs were routinely obtained after surgery to evaluate the osteotomy site and fixation position. After a 3-day latency period, transverse tibial distraction was initiated at a rate of 0.25 mm every 6 h. Patients continued distraction after discharge, consisting of lateral distraction for 10 days followed by medial distraction for another 10 days to restore the bone block to its original position. Radiographic examinations were performed during the second and fourth weeks of distraction to confirm bone block position.

All patients received standardized postoperative wound management, including daily dressing changes, diluted povidone-iodine disinfection, drainage with ethacridine-soaked gauze, and immobilization with short-leg casts or braces for protection and offloading. Partial weight-bearing assisted by walking aids was permitted during the early postoperative period because of the stability provided by the external fixation device. The external fixation frame was removed approximately 4 weeks after distraction.

Patients with abnormal ABI values (<0.9 or >1.4) and imaging-confirmed vascular calcification or stenosis who did not undergo percutaneous transluminal angioplasty received individualized medical therapy for circulation improvement and thrombosis prevention. Glycemic control was maintained through insulin injection or oral hypoglycemic agents, targeting fasting blood glucose of 6–8 mmol/L and 2-h postprandial glucose of 8–10 mmol/L.

### 2.3 Data collection and outcome definition

The detailed study workflow is shown in Figure 1. According to Wagner classification, patients were categorized into non-severe DFU (Wagner grades 2–3) and severe DFU (Wagner grades 4–5) groups. Baseline demographic characteristics, clinical characteristics, and laboratory parameters reflecting nutritional, inflammatory, and immune status were retrospectively collected. Postoperative laboratory measurements obtained at 1 month after TTT were also collected. For patients with multiple hospital admissions due to DFU, only data from the first admission were included. The primary endpoint was time to complete wound healing, defined as complete epithelialization without exudation and confirmed by the same experienced clinician. Patients were followed from TTT operation until complete wound healing or for a maximum of 12 months. Patients without complete healing at 12 months were administratively censored, while death and amputation before healing were considered potential competing events. Follow-up information was obtained through outpatient visits, telephone interviews, or electronic communication.

**Figure 1:**
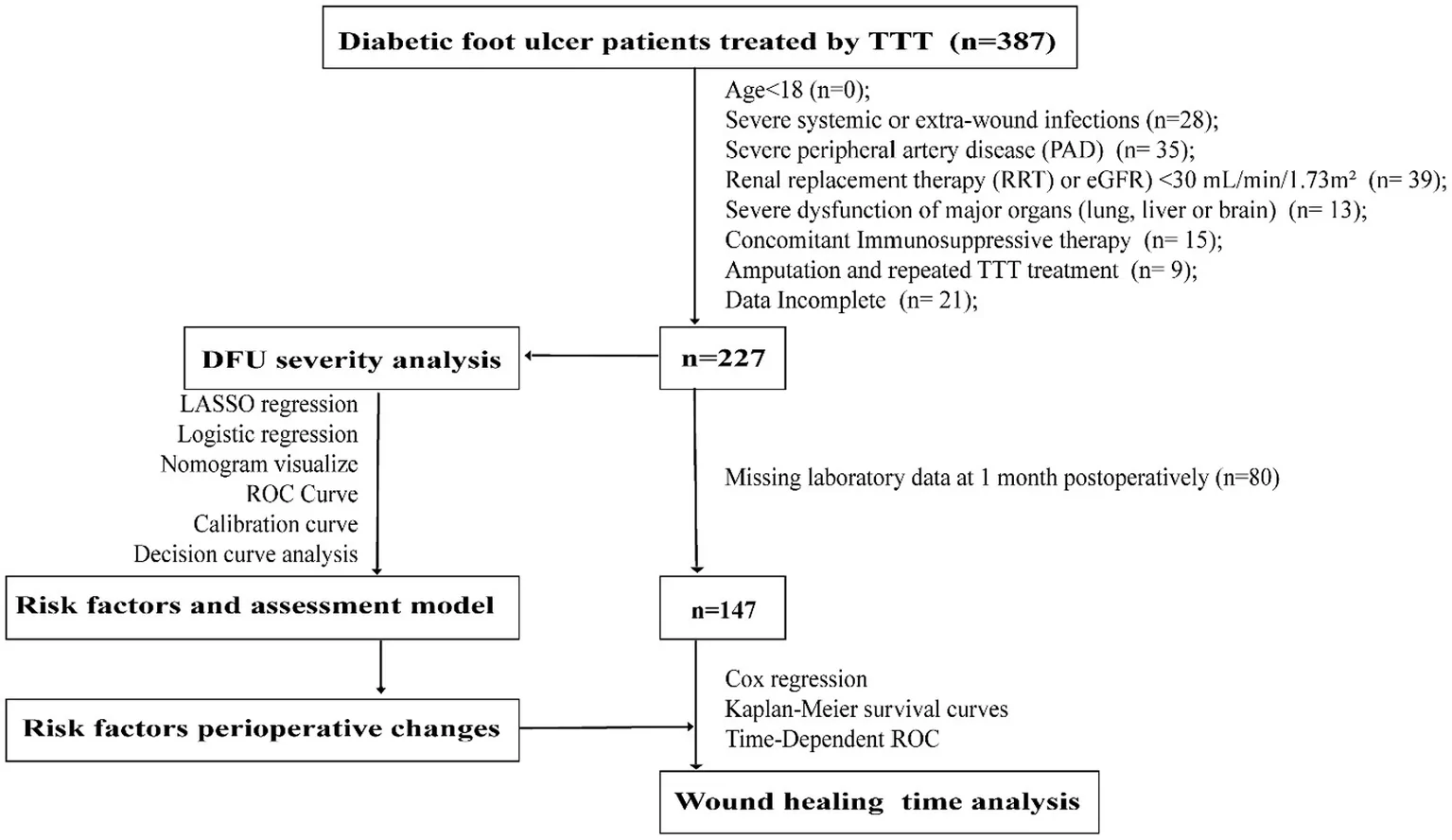
Diagram of research procedure. The study design comprised two analytical phases. First, 227 patients were included to identify factors associated with DFU severity. Nutritional–immune indicators were screened using LASSO regression, followed by multivariable logistic regression, and a nomogram was developed for severity assessment. Second, 147 patients with available postoperative 1-month laboratory data were included for wound healing analysis. Cox regression was performed to evaluate the association between nutritional–immune changes and wound healing time after TTT, with Kaplan–Meier analysis, sensitivity analyses, and time-dependent ROC analysis used for further evaluation.

BMI was calculated as weight (kg) divided by height squared (m^2^). PNI was calculated as 10 × serum albumin (g/dL) + 0.005 × peripheral blood lymphocyte count (/mm^3^) (18). ANLR was defined as ALB divided by the neutrophil-to-lymphocyte ratio (NLR) (13). The CONUT score was determined based on ALB, lymphocyte count, and total cholesterol (19). The change of PNI (ΔPNI) and ANLR (ΔANLR) was defined as postoperative minus preoperative values.

### 2.4 Statistical analysis

Baseline characteristics were analyzed in 227 patients included in the DFU severity analysis. Among them, 147 patients with complete postoperative 1-month laboratory measurements were included in the wound healing time analysis. Continuous variables with normal distribution were presented as mean ± standard deviation (SD), whereas non-normally distributed variables were presented as median with interquartile range (IQR). Categorical variables were presented as numbers and percentages. Comparisons between groups were performed using Student’s *t*-test for normally distributed continuous variables, Mann–Whitney *U* test for non-normally distributed continuous variables, and chi-square test for categorical variables.

### 2.5 Missing data handling

In the data preprocessing phase, the missing data patterns for each variable were first assessed. The results indicated that many variables had a missing data rates below 5% (the missing rate was defined as the proportion of missing observations in a variable relative to its total observations). Missing data were assumed to be missing at random (MAR). The missing data rates for each variable, are as follows: ABI (11,4.85%), eGFR (2,0.88%), Duration of diabetes (3,1.32%), BMI (6,2.64%), prealbumin (PA) (5,2.20%), Total cholesterol (4,1.76%), retinol-binding protein (RBP) (9,3.96%), 25(OH)D (8,3.52%) and CD4/CD8 ratio (9, 3.96%). Missing baseline covariates in both the DFU severity analysis (*n* = 227) and wound healing analysis (*n* = 147) were imputed using a random forest multiple imputation algorithm. Five imputed datasets were generated and combined according to Rubin’s rules to obtain final parameter estimates and standard errors. Patients without available postoperative 1-month PNI measurements were not included in the ΔPNI-based prognostic analysis because ΔPNI could not be calculated. Regression models involving baseline covariates were performed using imputed datasets.

### 2.6 Analysis of DFU severity

Univariable logistic regression analyses were first performed to assess the unadjusted associations between candidate variables and DFU severity. LASSO regression was subsequently applied for candidate variable selection. The penalty parameter was determined using 10-fold cross-validation, and λmin, corresponding to the minimum mean cross-validated error, was selected to retain potentially relevant variables for subsequent multivariable analysis. Variables retained by LASSO were then entered into a multivariable logistic regression model to identify factors independently associated with DFU severity. The discriminatory performance of the independently associated factors was assessed using receiver operating characteristic (ROC) curve analysis and the area under the curve (AUC). A combined model and corresponding nomogram were constructed based on these factors. The incremental value of the combined model over the individual indicator models was evaluated using nested likelihood-ratio tests, change in AUC (ΔAUC) with DeLong tests, net reclassification improvement (NRI), and integrated discrimination improvement (IDI). Internal validation was performed using bootstrap resampling, with estimation of the optimism-corrected AUC. Model calibration and clinical net benefit were assessed using calibration curves and decision curve analysis (DCA).

### 2.7 Analysis of wound healing time

Cox proportional hazards regression models were used to evaluate factors associated with wound healing time. The proportional hazards assumption was assessed using Schoenfeld residuals. To evaluate the robustness of the association between ΔPNI and wound healing time, sequential Cox models were constructed. Model 1 was an unadjusted model. Model 2 was adjusted for age, sex, smoking, and alcohol consumption. Model 3 was further adjusted for BMI, diabetes duration, HbA1c, ABI, eGFR, PA, and CRP. DFU severity was treated as a stratification factor in all models due to violation of the proportional hazards assumption. Covariates were selected based on clinical relevance and previous evidence. Sensitivity analysis was performed by identifying upper outliers of ΔPNI using the interquartile range (IQR) method and repeating Cox regression analysis after excluding these extreme values.

### 2.8 Survival analysis and time-dependent discrimination assessment

Kaplan–Meier curves with log-rank tests were used to compare wound healing trajectories according to ΔPNI and DFU severity. Time-dependent ROC curves were subsequently generated to evaluate the discrimination ability of the Cox model incorporating ΔPNI and DFU severity at 3, 6, and 12 months after TTT.

### 2.9 Statistical software

All statistical analyses were performed using IBM SPSS Statistics version 27.0 (IBM Corp., Armonk, NY, United States). R software (R Foundation for Statistical Computing, Vienna, Austria) was used for multiple imputation and modeling analyses. R packages included mice, glmnet, survival, timeROC, and dcurves. A two-sided *p*-value <0.05 was considered statistically significant.

## 3 Results

### 3.1 Baseline characteristics of patients with different DFU severity

Baseline characteristics are summarized in Table 1. A total of 227 patients with DFU who underwent TTT were included, including 108 patients (47.58%) with non-severe DFU (Wagner grades 2–3) and 119 patients (52.42%) with severe DFU (Wagner grades 4–5). No significant differences were observed between the two groups in demographic characteristics (age, sex, smoking, and alcohol consumption), diabetes-related parameters (diabetes duration and HbA1c), 25-hydroxyvitamin D levels, ABI, eGFR, or lymphocyte subset parameters (*p* > 0.05). Compared with non-severe DFU patients, severe DFU patients showed significantly higher inflammatory markers, including WBC, CRP and NLR. Whereas nutritional-related indicators, including BMI, hemoglobin, albumin, prealbumin, lymphocyte count, total cholesterol, serum calcium, and retinol-binding protein, were significantly lower (*p* < 0.05). In addition, PNI and ANLR were significantly decreased, while CONUT scores were increased in severe DFU patients (*p* < 0.05).

**Table 1:** Baseline characteristics of DFU patients stratified by DFU severity according to Wagner grades.

Variable	Overall (*n* = 227)	Non-severe DFU 108(47.58%)	Severe DFU 119(52.42%)	*t*/*z*/*χ*^2^	*p*-value
Sex					
Male	176(77.53%)	80(74.07%)	96(80.67%)	1.145	0.234
Female	51(22.47%)	28(25.93%)	23(19.33%)		
Age	62.00(55.00,69.25)	63.00(56.00,68.00)	61.00(55.00,70.00)	−0.316	0.752
Smoking status					
Yes	84(37.00%)	37(34.26%)	47(39.50%)	1.566	0.211
No	143(63.00%)	71(65.74%)	72(60.50%)		
Alcohol consumption					
Yes	68(29.96%)	29(26.85%)	39(32.77%)	2.256	0.133
No	159(70.04%)	79(73.15%)	80(67.23%)		
ABI	0.98(0.82,1.14)	1.01(0.84,1.14)	0.94(0.78,1.11)	−1.741	0.082
eGFR (mL/min/1.73 m^2^)	76.15(53.20,103.95)	79.80(55.95,106.20)	75.20 (45.35,99.55)	−0.395	0.693
Duration of diabetes (years)	7.0(3.0,11.0)	7.0(3.0,11.0)	7.0(3.0,12.0)	−0.127	0.899
HbA1c (%)	8.40(7.00,10.55)	8.50(7.33,10.58)	8.00(6.60,11.50)	−0.243	0.808
WBC (×10^9^/L)	10.10(7.50,13.44)	8.82(6.43,11.72)	10.83(8.90,14.32)	−3.498	<0.001
CRP (mg/L)	52.90(12.90,104.03)	19.50(6.13,65.22)	78.60(37.40,133.75)	−6.308	<0.001
Nutrition status					
BMI	22.72(20.54,24.98)	23.41(21.39,25.14)	22.04(19.56,24.76)	−2.855	0.004
Hb (g/L)	100.30(84.25,115.38)	108.40(93.45,120.75)	92.00(79.00,111.48)	−4.169	<0.001
Alb (g/L)	32.17 ± 6.12	35.04 ± 5.32	29.59 ± 5.65	7.440	<0.001
PA (mg/L)	130.50(81.40,193.65)	174.70(121.58,243.43)	100.30(60.70,146.90)	−6.614	<0.001
Total cholesterol (mmol/L)	3.94(3.27,4.82)	4.34(3.53,5.31)	3.81(3.24,4.63)	−2.643	0.008
RBP (mg/L)	30.80(20.34,44.36)	36.70(29.02,52.80)	23.95(15.91,37.55)	−5.224	<0.001
Serum Ca^2+^ (mmol/L)	2.14(2.00,2.28)	2.24(2.09,2.33)	2.07(1.97,2.17)	−5.563	<0.001
25(OH)D (nmol/L)	50.68(31.86,68.13)	53.26(32.45,68.68)	45.46(30.83,68.24)	−0.542	0.588
Lym (×10^9^/L)	1.61(1.18,2.11)	1.86(1.50,2.26)	1.29(1.00,1.77)	−4.439	<0.001
CD4/CD8 ratio	1.54(1.17,2.23)	1.57(1.17,2.25)	1.54(1.16,2.22)	−0.124	0.901
NLR	4.40(2.53,7.73)	2.870(1.97,5.13)	5.01(3.66,9.79)	−5.150	<0.001
ANLR	7.13(3.41,13.50)	12.42(7.63,18.07)	4.97(2.66,7.58)	−7.334	<0.001
CONUT	3.00(1.00,6.00)	2.00(0.00,4.00)	5.00(3.00,7.00)	−6.017	<0.001
PNI	40.38 ± 7.82	44.50 ± 6.93	36.68 ± 6.65	8.651	<0.001

Based on the Wagner’s grading of the selected patients, they were divided into 2 groups: Non-severe DFU group (Wagner’s 2–3) and Severe DFU group (Wagner’s 4–5). The baseline demographic and clinical characteristics of the study population are presented in the table.

Non-severe DFU (Wagner 2–3), Severe DFU (Wagner 4–5); ABI, ankle–brachial index; eGFR, estimated glomerular filtration rate (CKD-EPI); BMI, body mass index; HbA1c, glycated hemoglobin; WBC, white blood cell count; Hb, hemoglobin; Alb, albumin; PA, prealbumin; CRP, C-reactive protein; RBP, retinol-binding protein; 25(OH)D, 25-hydroxyvitamin D; Lym, Lymphocyte count; NLR, neutrophil to lymphocyte ratio; ANLR, albumin/neutrophil to lymphocyte ratio; CONUT, controlling nutritional status; PNI, prognostic nutritional index.

### 3.2 Nutritional–immune indicators associated with DFU severity

Univariable logistic regression analysis was first performed to evaluate the associations between candidate variables and DFU severity (Supplementary Table 1). LASSO regression was subsequently applied for further variable selection. Ten-fold cross-validation identified λmin = 0.065 as the optimal penalty parameter, and five candidate variables, including Hb, CRP, prealbumin, ANLR, and PNI, were retained for subsequent multivariable analysis (Figures 2A,B). All selected variables showed VIF values <5, indicating no significant multicollinearity (Supplementary Table 2). Multivariable logistic regression analysis demonstrated that only ANLR (OR = 0.993, 95% CI: 0.987–0.999, *p* = 0.020) and PNI (OR = 0.909, 95% CI: 0.853–0.969, *p* = 0.004) remained independently associated with DFU severity (Table 2). To facilitate interpretation of the ANLR effect magnitude, we additionally expressed the association on a standardized scale. Each 1-SD increase in ANLR (SD = 8.87) was associated with lower odds of severe DFU (OR = 0.940, 95% CI: 0.890–0.991, *p* = 0.020) (Supplementary Table 3). ROC analysis showed that ANLR had an AUC of 0.797 (95% CI: 0.731–0.858), with an optimal cut-off of 8.91, sensitivity of 0.815, and specificity of 0.737, PPV of 0.717, and NPV of 0.782. PNI showed an AUC of 0.805 (95% CI: 0.747–0.866), with an optimal cut-off of 42.78, sensitivity of 0.814, and specificity of 0.684, PPV of 0.770, and NPV of 0.717 (Figure 2C). Based on the multivariable logistic regression results, a combined model incorporating PNI and ANLR was constructed and visualized as a nomogram. The nomogram incorporating ANLR and PNI showed improved AUC of 0.825 (95% CI: 0.756–0.893). The optimal cut-off was 0.539, with sensitivity of 0.821, specificity of 0.739, PPV of 0.780, and NPV of 0.785 (Figures 2D,E). Incremental analyses suggested that the combined model may offer additional discriminatory value over the individual indicators, although the strength of evidence differed between comparisons with PNI and ANLR (Supplementary Table 4). Bootstrap internal validation yielded a corrected AUC of 0.819 (95% CI: 0.754–0.891), close to the apparent AUC of 0.825 (Supplementary Figure 1A). Calibration analysis demonstrated good agreement between predicted and observed outcomes, and DCA showed higher estimated net benefit across a wide range of threshold probabilities (Supplementary Figures 1B,C).

**Figure 2:**
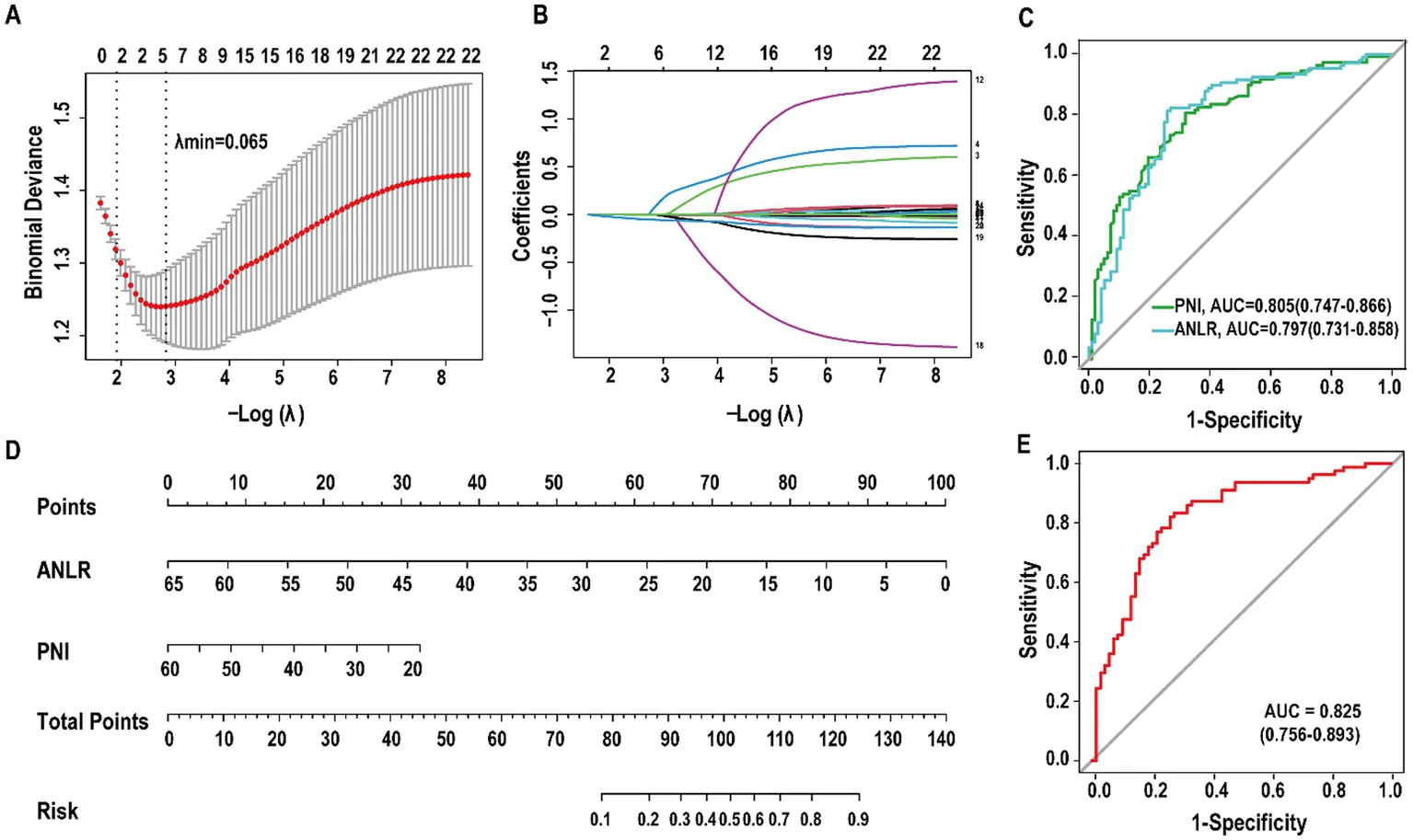
Identification of nutritional–immune indicators associated with DFU severity and development of a severity assessment model. **(A)** Ten-fold cross-validation curve identifying the optimal penalty parameter (λ) in LASSO regression based on minimum binomial deviance. **(B)** LASSO coefficient profiles showing variable selection with increasing penalty strength, with five candidate variables retained for multivariable analysis. **(C)** ROC curves demonstrating the discriminative performance of PNI and ANLR for distinguishing severe from non-severe DFU. The AUCs were 0.805 (95% CI, 0.747–0.866) for PNI and 0.797 (95% CI, 0.731–0.858) for ANLR. **(D)** Nomogram developed using independent factors (PNI and ANLR) from multivariable logistic regression for individualized DFU severity assessment. **(E)** ROC curve of the combined PNI–ANLR model, showing the discriminative performance of the combined model (AUC = 0.825; 95% CI, 0.756–0.893).

**Table 2:** Multivariable logistic regression analysis identifying factors associated with DFU severity.

Variable	Coefficient	Wald	*P*-value	OR	95%CI low	95%CI up
Hb	−0.004	0.119	0.730	0.996	0.973	1.020
CRP	0.003	0.295	0.587	1.003	0.993	1.013
PA	−0.006	1.498	0.221	0.994	0.984	1.004
ANLR	−0.007	5.392	0.020	0.993	0.987	0.999
PNI	−0.095	8.199	0.004	0.909	0.853	0.969

Hb, CRP, PA, ANLR, and PNI were selected as candidate predictors by LASSO regression analysis, and multivariable logistic regression demonstrated that only ANLR and PNI remained independent risk factors.

Hb, hemoglobin; CRP, C-reactive protein; PA, prealbumin; ANLR, albumin/neutrophil to lymphocyte ratio; PNI, prognostic nutritional index.

### 3.3 Dynamic changes in nutritional–immune indicators after TTT

Among the 227 patients, 147 patients with complete postoperative 1-month laboratory data were included for dynamic analysis. Baseline characteristics between patients with and without complete postoperative laboratory measurements showed no significant differences (Supplementary Table 5). Compared with baseline values, both PNI and ANLR significantly increased at 1 month after TTT (*p* < 0.05) (Supplementary Table 6 and Figure 3), indicating dynamic changes in nutritional–immune status during the postoperative period.

**Figure 3:**
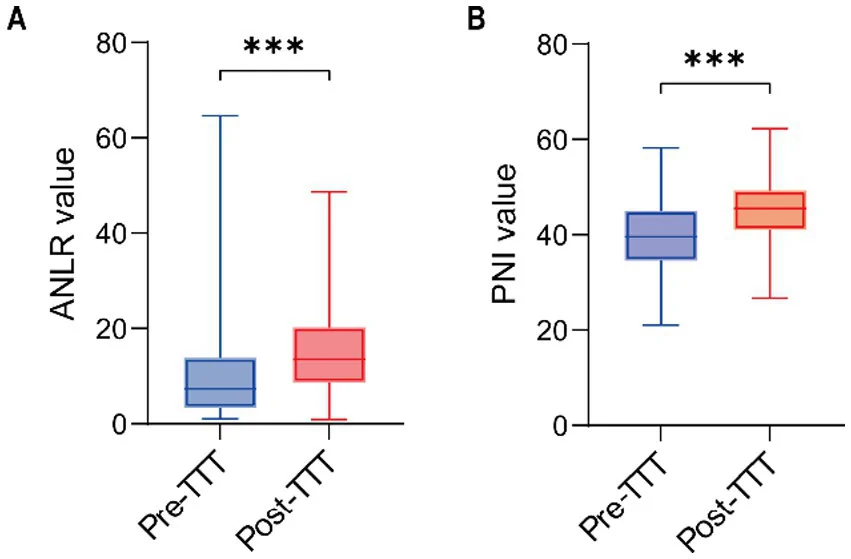
Changes in ANLR and PNI before and 1 month after TTT surgery. Box plots show the distribution of ANLR **(A)** and PNI **(B)** at preoperative and 1-month postoperative time points. Statistical significance was assessed using paired t-test or Wilcoxon signed-rank test according to data distribution (***p < 0.001).

### 3.4 Association between ΔPNI and wound healing time after TTT

Cox proportional hazards regression was performed to identify factors associated with wound healing time. Although baseline PNI and ANLR were associated with DFU severity, neither was significantly associated with postoperative wound healing time (Supplementary Table 7). Therefore, we further evaluated the relationship between dynamic nutritional–immune changes and wound healing. Univariable Cox analysis showed that age, DFU severity, and ΔPNI were associated with wound healing time (*p* < 0.05). In contrast, ΔANLR was not significantly associated with wound healing time (*p* = 0.177) (Supplementary Table 7). Variables showing significant associations in the univariable analysis were subsequently considered for multivariable Cox regression analysis to identify independent factors associated with wound healing time. In the initial multivariable Cox regression analysis, DFU severity and ΔPNI remained independently associated with wound healing time (Table 3). Higher DFU severity was associated with slower wound healing (HR = 0.673, 95% CI: 0.473–0.960, *p* = 0.029), and higher ΔPNI was associated with a higher hazard of complete wound healing (HR = 1.027, 95% CI: 1.004–1.048, *p* = 0.021). Schoenfeld residual analysis showed that ΔPNI satisfied the proportional hazards assumption (*χ*^2^ = 0.631, *p* = 0.427), whereas DFU severity showed mild deviation (*χ*^2^ = 3.976, *p* = 0.046). The global test indicated no significant violation of the proportional hazards assumption (*χ*^2^ = 5.128, *p* = 0.077). Accordingly, DFU severity was subsequently treated as a stratification factor in the Cox model. After stratification by DFU severity, ΔPNI remained significantly associated with wound healing time (HR = 1.030, 95% CI: 1.008–1.053, *p* = 0.008), supporting the consistency of this association (Supplementary Table 8 and Supplementary Figure 2).

**Table 3:** Initial multivariable Cox proportional hazards regression analysis of factors associated with wound healing time after TTT.

Variable	Coefficient	Wald	*p*-value	HR	95%CI low	95%CI up
Age	−0.012	1.509	0.130	0.988	0.969	1.007
Severity status	−0.395	4.780	0.029	0.673	0.473	0.960
ΔPNI	0.025	5.334	0.021	1.027	1.004	1.048

DFU severity was initially included as a covariate in the conventional Cox model. DFU severity was included as a stratification variable due to violation of the proportional hazards assumption. Multivariable Cox analysis showed that ΔPNI were significantly associated with wound healing.

Severity status (non-severe status which classified as Wagner’s 2–3; Severe status which classified as Wagner’s 4–5); ΔPNI was calculated as postoperative PNI minus preoperative PNI.

### 3.5 Sensitivity analyses

To assess the robustness of the association between ΔPNI and wound healing time under different levels of covariate adjustment, sequential Cox regression models were constructed. ΔPNI was analyzed as a continuous variable and evaluated per 1-unit increase and per 1-SD increase. Model 1 was a crude model. Model 2 was adjusted for age, sex, smoking status, and alcohol consumption. Model 3 was further adjusted for diabetes duration, HbA1c, BMI, ABI, eGFR, prealbumin, and CRP. DFU severity was incorporated as a stratification factor in all models because it violated the proportional hazards assumption (Table 4). Considering that extremely large positive ΔPNI values may be influenced by perioperative interventions, upper outliers were identified using the IQR method and excluded for sensitivity analysis. After exclusion of these extreme values, the association between ΔPNI and wound healing time remained unchanged (Table 5), suggesting that the observed association was not driven by extreme observations. Next, among the 147 patients included in the wound-healing analysis, 99 (67.35%) received albumin supplementation, whereas 48 (32.65%) did not. Among those who received albumin, the median cumulative dose was 60 g (IQR, 40–90 g). Additional sensitivity analyses showed that the association between ΔPNI and subsequent wound healing remained generally consistent after further adjustment for cumulative perioperative albumin dose (HR = 1.036, 95% CI: 1.007–1.066, *p* = 0.014), with similar results observed when albumin supplementation status was included instead of cumulative dose. After additional adjustment for WBC and CRP at 1 month as available indicators of concurrent systemic inflammatory status, the association was modestly attenuated but remained statistically significant (HR = 1.031, 95% CI: 1.001–1.061, *p* = 0.039) (Supplementary Table 9).

**Table 4:** Sequentially adjusted severity-stratified Cox regression models for the association between ΔPNI and subsequent wound healing.

Indicator	Model 1 HR (95% CI)	*p*-value	Model 2 HR (95% CI)	*P*-value	Model 3 HR (95% CI)	*P*-value
ΔPNI, per 1-unit increase	1.030(1.008–1.053)	0.008	1.029(1.005–1.052)	0.015	1.037(1.009–1.065)	0.009
ΔPNI, per 1-SD increase	1.246(1.060–1.466)	0.008	1.233(1.041–1.459)	0.015	1.308(1.069–1.600)	0.009

Model 1: crude model including ΔPNI.

Model 2: adjusted for age, sex, smoking status, and alcohol consumption.

Model 3: further adjusted for diabetes duration, HbA1c, ABI, BMI, eGFR, CRP, and PA.

DFU severity was incorporated as a stratification factor in all models because Schoenfeld residual testing indicated a mild violation of the proportional hazards assumption (*p* = 0.046). ΔPNI was analyzed as a continuous variable and reported per 1-unit increase and per 1-SD increase (SD = 7.43). HR > 1 indicates a higher hazard of achieving wound healing.

**Table 5:** Sequentially adjusted severity-stratified Cox regression models for the association between ΔPNI and subsequent wound healing after exclusion of extreme ΔPNI values.

Indicator	Model 1 HR (95% CI)	*P*-value	Model 2 HR (95% CI)	*P*-value	Model 3 HR (95% CI)	*P*-value
ΔPNI, per 1-unit increase	1.039(1.011–1.066)	0.005	1.038(1.010–1.067)	0.007	1.049(1.015–1.084)	0.005
ΔPNI, per 1-SD increase	1.325(1.088–1.613)	0.005	1.321(1.079–1.619)	0.007	1.424(1.113–1.820)	0.005

Model 1: crude model including ΔPNI.

Model 2: adjusted for age, sex, smoking status, and alcohol consumption.

Model 3: further adjusted for diabetes duration, HbA1c, ABI, BMI, eGFR, CRP, and PA.

DFU severity was incorporated as a stratification factor in all models due to violation of the proportional hazards assumption. ΔPNI was analyzed as a continuous variable and reported per 1-unit increase and per 1-SD increase (SD = 7.43). HR > 1 indicates a higher hazard of achieving wound healing. Sensitivity analysis was performed after excluding patients with extreme ΔPNI values using the 1.5 × IQR rule.

### 3.6 Kaplan–Meier analysis

Among the 147 patients included in the wound-healing analysis, 141 (95.9%) achieved complete wound healing within 12 months and were considered healing events. The remaining 6 patients (4.1%) had not achieved complete wound healing by 12 months and were administratively censored at that time. The median observation time from TTT to complete wound healing or censoring was 2.93 months (IQR, 2.10–3.83 months). No deaths or amputations occurred before complete wound healing during the observation period. Patients were categorized into high and low ΔPNI groups according to the median ΔPNI value (5.425). Kaplan–Meier analysis showed that severe DFU patients had significantly higher cumulative non-healing probability than non-severe DFU patients (log-rank *p* = 0.045). Similarly, patients with lower ΔPNI showed slower wound healing compared with those with higher ΔPNI (log-rank *p* = 0.018) (Figure 4A).

**Figure 4:**
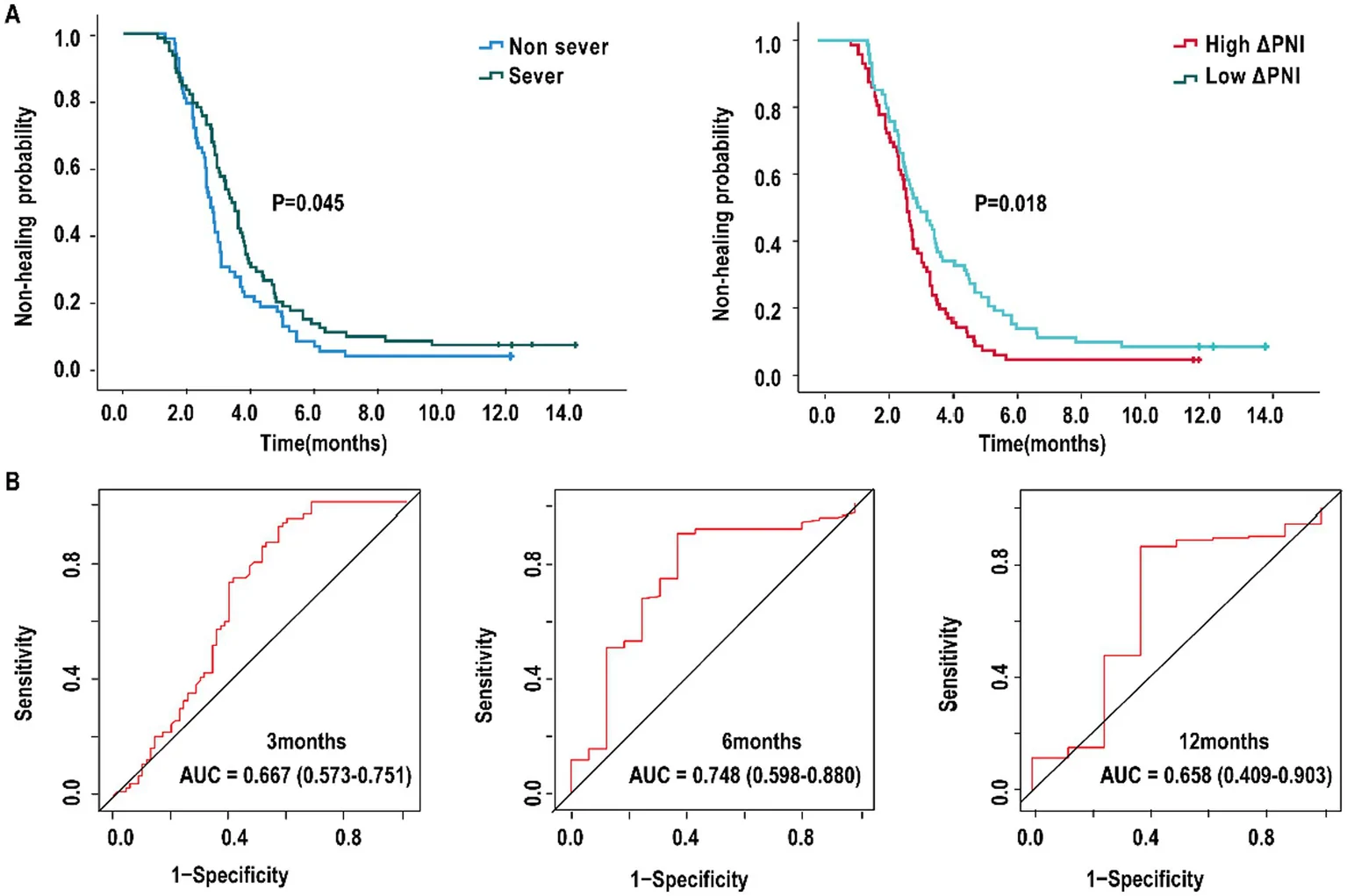
Kaplan–Meier analysis and time-dependent ROC evaluation of wound healing after TTT. **(A)** Kaplan–Meier curves illustrating differences in wound healing time according to DFU severity and ΔPNI levels. Patients with severe DFU or lower ΔPNI showed significantly different wound healing probabilities (log-rank *p* = 0.045 and 0.018). **(B)** Time-dependent ROC curves assessing the discrimination ability of the Cox model incorporating ΔPNI and DFU severity for wound healing outcomes at 3, 6, and 12 months after TTT.

### 3.7 Time-dependent ROC analysis

The linear predictor derived from the primary severity-stratified Cox model was used to evaluate time-dependent discrimination ability. The model showed variable discrimination performance over follow-up. Time-dependent ROC analysis showed AUCs of 0.667 (95% CI: 0.573–0.751), 0.748 (95% CI: 0.598–0.880), and 0.658 (95% CI: 0.409–0.903) at 3, 6, and 12 months, respectively. The highest discriminatory performance was observed at approximately 6 months, whereas the wide 95% CI at 12 months indicated considerable uncertainty in the longer-term estimate (Figure 4B).

Finally, to visually illustrate the association between ΔPNI and wound healing trajectories, representative healing courses of patients with Wagner grade 4 DFU were presented. Patients with higher ΔPNI showed a relatively faster wound recovery trend after TTT, whereas those with lower ΔPNI demonstrated slower recovery trajectories (Figure 5).

**Figure 5:**
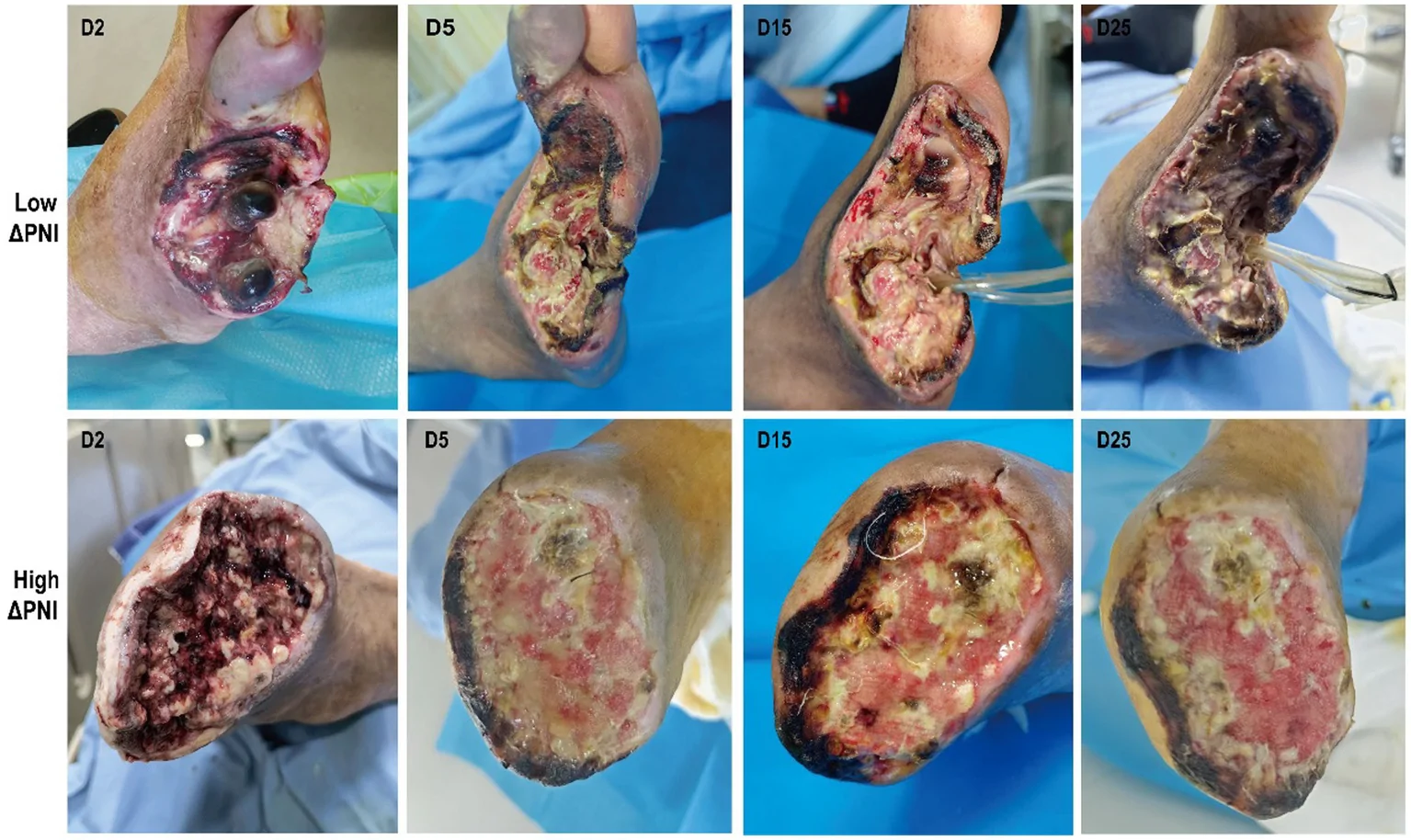
Representative wound changes 1 month after TTT in Wagner Grade 4 patients according to different ΔPNI levels. By postoperative day 25, wounds in the low PNI improvement group (top row) deteriorated with increasing necrosis; despite repeated debridement and bull-nose drain insertion, the wounds enlarged with extensive secondary necrosis and lacked healing progression. Conversely, the high PNI improvement group (bottom row) demonstrated resolving edema, robust granulation tissue growth, limited secondary necrosis, and progressive wound contraction consistent with healing.

## 4 Discussion

The development, progression, and postoperative recovery of DFU are complex processes influenced by multiple factors, including local tissue damage, infection, vascular function, and systemic metabolic and immune status. Previous studies investigating the prognosis of DFU patients treated with tibial transverse transport (TTT) have mainly focused on local factors, such as vascular status, tissue oxygenation, and inflammatory indicators. However, the relationship between host nutritional–immune status as well as their dynamic changes and wound recovery after TTT remains insufficiently explored. To our knowledge, this study is among the first to investigate the association between nutritional–immune indicators and their dynamic changes with wound healing time in DFU patients undergoing TTT, providing new insights into the heterogeneity of recovery trajectories after TTT treatment.

The major findings of this study were as follows. First, patients with severe DFU exhibited more pronounced systemic inflammatory responses and nutritional–immune disturbances, with PNI and ANLR independently associated with DFU severity. Second, although baseline PNI and ANLR reflected the initial disease burden, neither was significantly associated with wound healing time after TTT. In contrast, the change in PNI at 1 month after surgery (ΔPNI) was independently associated with subsequent wound healing time and remained stable after adjustment for DFU severity and multiple clinical factors. These findings suggest that baseline nutritional–immune status may primarily reflect disease severity, whereas dynamic changes in nutritional–immune status during treatment may provide additional information related to patient recovery.

DFU is not solely determined by local factors such as vascular impairment, neuropathy, and infection severity. Increasing evidence indicates that systemic inflammation, immune dysfunction, and insufficient nutritional reserves also contribute to the development and progression of chronic wounds (20). In addition to vascular disease, neuropathy, and infection severity, various clinical characteristics, including male sex, smoking, longer diabetes duration, and poor glycemic control reflected by elevated glycated hemoglobin levels, have been associated with DFU severity and prognosis (21, 22). Comorbidities such as diabetic retinopathy, chronic kidney disease, and heart failure have also been reported to influence DFU outcomes (23). Furthermore, nutritional abnormalities, including lower albumin and serum calcium levels, have been associated with disease progression (24). Prediction models incorporating multiple clinical factors have demonstrated improved performance for DFU severity assessment (25). Therefore, this study comprehensively evaluated multiple inflammatory, nutritional, and immune-related indicators, including prealbumin (PA), serum 25-hydroxyvitamin D, and retinol-binding protein (RBP), which, all have recently been associated with DFU severity and prognosis (26, 27). In addition, composite nutritional–immune indicators, including PNI, ANLR, and CONUT were included to compare the value of different dimensions of indicators in assessing DFU severity and outcomes.

In this study, although several nutritional indicators, including BMI, hemoglobin, albumin, prealbumin, retinol-binding protein (RBP) and lymphocyte count, as well as inflammatory indicators such as WBC, CRP and NLR, differed significantly between severe and non-severe DFU groups, only ANLR and PNI remained independently associated with DFU severity in multivariable Logistic regression analysis. Although BMI differed significantly between the two severity groups, it was not retained by the LASSO procedure when nutritional, inflammatory, and immune-related variables were considered simultaneously, indicating that its association was not retained in the penalized variable-selection procedure. Notably, the per-unit OR for ANLR was close to unity, this partly reflected the variable scale. When expressed per 1-SD increase, higher ANLR was associated with lower odds of severe DFU. Nevertheless, the magnitude of this association remained modest, suggesting that the value of ANLR as a standalone indicator may be limited.

Despite their overlapping components, particularly the shared inclusion of albumin and lymphocyte-related information, both PNI and ANLR remained independently associated with DFU severity without evidence of substantial multicollinearity. Incremental analyses suggested that combining PNI and ANLR may provide additional information beyond either indicator alone, although the evidence was more consistent when the combined model was compared with PNI than with ANLR alone. The bootstrap-corrected AUC (0.819) was close to the apparent AUC (0.825), suggesting limited optimism. However, the lack of comparison with a prespecified clinical-variable model remains an important limitation, and the incremental and clinical value of the combined PNI–ANLR model requires confirmation in larger, independent cohorts. Overall, these findings suggest that composite nutritional–immune indicators may provide a broader representation of host nutritional and immune-inflammatory status than individual laboratory measures, supporting their potential value in the assessment of DFU severity (28).

The TTT has become an important limb-salvage strategy for patients with complex DFU and is considered as a reliable salvage treatment when conventional therapies fail (17, 29). However, whether TTT provides consistent benefits for all DFU patients remains insufficiently investigated. Moreover, due to the relatively prolonged treatment course, reliable indicators reflecting patient recovery during TTT remain lacking. Previous studies have demonstrated that preoperative local factors, such as foot temperature and transcutaneous oxygen pressure, are associated with TTT outcomes (8). Although these parameters reflect wound perfusion status, they do not capture the influence of systemic organ function and metabolic status on disease recovery. In addition, inflammatory indicators, including higher NLR, MNR, and PLR, have been associated with poorer outcomes after TTT (15, 16). However, these studies mainly focused on preoperative inflammatory and immune status and did not adequately evaluate dynamic changes in overall patient conditions during treatment. Previous research has also shown that preoperative PA levels were associated with in-hospital mortality in patients with severe DFU undergoing TTT, indicating the importance of nutritional status in this population (30). In the present study, although baseline PNI and ANLR were associated with DFU severity, neither was significantly associated with wound healing time after TTT. In contrast, greater ΔPNI at 1 month after surgery was independently associated with subsequent wound healing. Time-dependent ROC analysis showed the strongest discriminatory performance at approximately 6 months, whereas the wide confidence interval at 12 months indicated substantial uncertainty regarding longer-term discrimination. Overall, these findings suggest that dynamic changes in nutritional–immune status during the early postoperative period may be more closely associated with subsequent wound-healing trajectories than baseline nutritional–immune status alone.

It should be emphasized that ΔPNI, as a postoperative dynamic indicator, should not be interpreted as a conventional pretreatment predictive factor. The change in PNI within 1 month after surgery may be influenced by multiple factors, including infection control, wound management, nutritional intake, and perioperative supportive interventions (7, 31). Therefore, a higher ΔPNI may reflect better overall recovery status rather than directly contribute to accelerated wound healing. In the present study, none of the patients achieved complete wound healing within 1 month after surgery, which may partially reduce the potential influence of immortal-time bias. However, given the observational nature of this study, reverse causality cannot be completely excluded. Although perioperative albumin supplementation, WBC and CRP at 1 month were additionally considered in sensitivity analyses to partially account for ΔPNI from albumin infusion and concurrent systemic inflammatory status, quantitative changes in wound status and standardized assessments of local infection control were not systematically available because of the retrospective design. Furthermore, the absence of a non-TTT treatment control group prevents us from determining whether TTT directly induced PNI improvement to promoted wound healing. Therefore, the biological significance of ΔPNI requires further clarification. ΔPNI may be more appropriately interpreted as a dynamic marker reflecting integrated response of nutritional–immune status during treatment rather than a definitive causal factor.

Wound healing in DFU is a multifactorial process, and nutritional–immune status should be considered a complementary component rather than a replacement for traditional clinical factors. In this study, ABI and eGFR, representing vascular status and renal function, respectively, were included as potential influencing factors; however, neither showed a significant association with DFU severity or wound healing time after TTT. This finding may be related to the inherent limitations of these indicators and the specific characteristics of TTT treatment. ABI mainly reflects large-vessel function, whereas diabetes-related vascular calcification, particularly in patients with renal dysfunction, may impair its accuracy in evaluating actual vascular status (32, 33). In addition, TTT is considered to exert therapeutic effects mainly through improving microcirculation and promoting angiogenesis (34, 35), which may not be fully reflected by ABI-related macrovascular changes. Similarly, the association between eGFR and DFU outcomes may vary depending on calculation methods, patient characteristics, and definitions of disease severity. Previous studies have suggested that mild renal impairment (eGFR 60–89 mL/min/1.73 m^2^) may not be consistently associated with DFU severity, delayed wound healing, or amputation events (36). Moreover, the relatively limited sample size in our study may have reduced the statistical power to detect potential associations between eGFR and clinical outcomes. Therefore, the lack of statistical significance for ABI and eGFR does not necessarily indicate limited clinical relevance, but rather suggests that single traditional indicators may be insufficient to fully explain wound recovery after TTT. In contrast, PNI and ANLR are derived from routinely available laboratory parameters with good accessibility and reproducibility, suggesting their potential value as auxiliary indicators for DFU severity assessment. Meanwhile, ΔPNI may serve as a dynamic marker reflecting recovery status during treatment rather than an independent basis for clinical decision-making. Future studies integrating wound characteristics, vascular status, infection control, and metabolic parameters may facilitate the development of comprehensive evaluation systems for individualized management.

This study has several limitations. First, this was a single-center retrospective study with a relatively limited sample size, and further validation in multicenter prospective cohorts is required. Second, vascular status in DFU patients was primarily assessed using ABI, while other comprehensive vascular parameters, including foot temperature, transcutaneous oxygen pressure, and rest pain, were not available, which may have resulted in residual confounding. Third, due to incomplete postoperative laboratory data, only 147 patients were included in the wound healing analysis. Although baseline characteristics showed no significant differences between included and excluded patients, and only three variables were included in the multivariable Cox model, potential selection bias cannot be completely excluded. Fourth, wound healing outcomes were mainly obtained through outpatient follow-up, telephone interviews, or WeChat follow-up. Although all assessments were performed by the same experienced physician and the outcome definition was clearly established, some degree of measurement error may remain. Finally, given the exploratory design and the number of indicators evaluated, multiple-comparison adjustment could not be fully addressed, and residual confounding from unmeasured factors cannot be excluded. These limitations warrant cautious interpretation and further validation in prospective multicenter studies with dynamic monitoring of nutritional–immune changes and subsequent wound healing.

## 5 Conclusion

In conclusion, PNI and ANLR were independently associated with DFU severity, suggesting their potential value as complementary indicators for preoperative risk stratification. Although baseline PNI and ANLR were not associated with postoperative wound healing time, improvement in PNI at 1 month after TTT (ΔPNI) was associated with subsequent wound healing and may represent an integrated response of nutritional–immune status during the treatment course. Given the retrospective design and potential influence of perioperative interventions, these findings should be interpreted cautiously. Further prospective multicenter studies are needed to validate the clinical significance of nutritional–immune changes in DFU wound healing.

## Data Availability

The original contributions presented in the study are included in the article/Supplementary material, further inquiries can be directed to the corresponding author.
