# Supplementary material for: Association of nutritional–immune indicators and their dynamic changes with disease severity and wound healing in diabetic foot ulcers treated by tibial transverse transport: a retrospective study
Source: Front Nutr. 2026 Oct 8;13:1863436. doi: 10.3389/fnut.2026.1863436 (PMC13687399; doi:10.3389/fnut.2026.1863436)
Supplement: Supplementary file 1 [file supplementary_file_1.docx]

Supplementary Material

**Supplementary Table 1. Univariable logistic regression analysis of factors associated with DFU severity.**

| **Variable** | **Coefficient** | **Wald** | **p-value** | **OR** | **95%CI low** | **95%CI up** |
| --- | --- | --- | --- | --- | --- | --- |
| Sex | -0.379 | 1.407 | 0.236 | 0.685 | 0.366 | 1.281 |
| Age | -0.003 | 0.032 | 0.857 | 0.997 | 0.966 | 1.030 |
| Smoking status | 0.404 | 1.425 | 0.233 | 1.497 | 0.772 | 2.904 |
| Alcohol consumption | 0.611 | 2.873 | 0.090 | 1.843 | 0.909 | 3.736 |
| ABI | -1.270 | 2.599 | 0.107 | 0.281 | 0.060 | 1.315 |
| eGFR | -0.002 | 0.202 | 0.653 | 0.998 | 0.987 | 1.008 |
| Duration of diabetes | 0.006 | 0.101 | 0.750 | 1.006 | 0.968 | 1.046 |
| HbA1c | 0.037 | 0.313 | 0.576 | 1.038 | 0.911 | 1.182 |
| WBC | 0.097 | 7.164 | 0.007 | 1.102 | 1.026 | 1.183 |
| CRP | 0.011 | 21.792 | <0.001 | 1.011 | 1.007 | 1.016 |
| Nutrition status |  |  |  |  |  |  |
| BMI | 0.002 | 0.355 | 0.551 | 1.002 | 0.996 | 1.007 |
| Hb | -0.024 | 12.623 | <0.001 | 0.977 | 0.964 | 0.989 |
| Alb | -0.176 | 37.528 | <0.001 | 0.839 | 0.793 | 0.887 |
| PA | -0.013 | 35.506 | <0.001 | 0.987 | 0.982 | 0.991 |
| Total cholesterol | -0.453 | 8.145 | 0.004 | 0.635 | 0.465 | 0.868 |
| RBP | -0.026 | 13.040 | <0.001 | 0.974 | 0.961 | 0.988 |
| Serum Ca^2+^ | -4.327 | 25.185 | <0.001 | 0.013 | 0.002 | 0.072 |
| 25(OH)D | -.004 | 0.190 | 0.663 | 0.996 | 0.979 | 1.014 |
| Lym (×10⁹/L) | -1.297 | 16.461 | <0.001 | 0.278 | 0.150 | 0.516 |
| CD4/CD8 ratio | 0.176 | 0.572 | 0.450 | 1.193 | 0.755 | 1.884 |
| NLR | 0.116 | 8.776 | 0.003 | 1.123 | 1.040 | 1.213 |
| ANLR | -0.191 | 38.193 | <0.001 | 0.826 | 0.778 | 0.878 |
| CONUT | 0.330 | 32.408 | <0.001 | 1.391 | 1.241 | 1.558 |
| PNI | -0.166 | 44.519 | <0.001 | 0.847 | 0.807 | 0.889 |

Abbreviations: Non-severe DFU (Wagner 2-3), Severe DFU (Wagner 4-5); ABI, ankle–brachial index; eGFR, estimated glomerular filtration rate(CKD-EPI); BMI, body mass index; HbA1c, glycated hemoglobin; WBC, white blood cell count; Hb, hemoglobin; Alb, albumin; PA, prealbumin; CRP, C-reactive protein; RBP, retinol-binding protein; 25(OH)D, 25-hydroxyvitamin D; Lym, Lymphocyte count; NLR, neutrophil to lymphocyte ratio; ANLR, albumin/neutrophil to lymphocyte ratio; CONUT, controlling nutritional status; PNI, prognostic nutritional index.

**Supplementary Table 2. Multicollinearity diagnostics of variables selected by LASSO regression.**

| **Variable** | **Variance Inflation Factor (VIF)** |
| --- | --- |
| **Hb** | 3.235 |
| **PA** | 2.417 |
| **CRP** | 1.574 |
| **PNI** | 1.047 |
| **ANLR** | 2.049 |

Abbreviations: Hb, hemoglobin; PA, Prealbumin; CRP, C-reactive protein; ANLR, albumin/neutrophil to lymphocyte ratio; PNI, prognostic nutritional index.

Before constructing the multivariate logistic regression model, we assessed the multicollinearity among the variables selected by LASSO regression. VIF values <5 indicated no significant multicollinearity among the selected variables.

**Supplementary Table 3. Association between ANLR and DFU severity expressed per unit and per 1-SD increase.**

| Variable | Coefficient | Wald | P value | OR | 95%CI low | 95%CI up |
| --- | --- | --- | --- | --- | --- | --- |
| ANLR | -0.007 | 5.392 | 0.020 | 0.993 | 0.987 | 0.999 |
| Per 1-SD increase | -0.062 | 5.392 | 0.020 | 0.940 | 0.890 | 0.991 |

ANLR was analyzed on both its original scale and a standardized scale. The standard deviation of ANLR was 8.87. ORs are presented per 1-unit and per 1-SD increase in ANLR. Both estimates were derived from the same multivariable logistic regression model including Hb, CRP, PA, ANLR, and PNI.

**Supplementary Table 4. Incremental value of the combined PNI–ANLR model compared with single-indicator models for DFU severity assessment.**

|  | **Likelihood-ratio test, P** | **ΔAUC (95% CI)** | **DeLong test, Z (P value)** | **NRI (95% CI), P value** | **IDI (95% CI), P value** |
| --- | --- | --- | --- | --- | --- |
| **Combined model vs. ANLR** | P=0.031 | 0.014 (-0.015-0.044) | Z = 0.952, P=0.341 | 0.384(0.067-0.700), P=0.018 | 0.027(0.001- 0.053), P=0.039 |
| **Combined model vs. PNI** | P<0.001 | 0.042 (0.002-0.082) | Z = 2.054, P=0.040 | 0.503(0.189-0.816), P=0.002 | 0.073(0.032- 0.113), P<0.001 |

Abbreviations: AUC, area under the receiver operating characteristic curve; ΔAUC, change in AUC; NRI, net reclassification improvement; IDI, integrated discrimination improvement; ANLR, albumin-to-neutrophil-to-lymphocyte ratio; PNI, prognostic nutritional index; CI, confidence interval.

**Supplementary Figure 1. Internal validation and performance assessment of the combined PNI–ANLR model for DFU severity.**

A. Bootstrap-corrected receiver operating characteristic curve of the combined PNI–ANLR model.
B. Calibration curve of the combined PNI–ANLR model.
C. Decision curve analysis of the combined PNI–ANLR model.

**Supplementary Table 5. Baseline characteristics of patients included and excluded from the prognostic analysis according to postoperative laboratory data availability.**

| **Variable** | **Unavailable postoperative data**  **(n = 80)** | **Available postoperative data**  **(n = 147)** | **t/z/χ2** | **p value** |
| --- | --- | --- | --- | --- |
| Sex |  |  |  |  |
| Male | 59(73.75%) | 117(79.59%) | -0.183 | 0.855 |
| Female | 21(26.25%) | 30(20.41%) |  |  |
| Age | 61.00(52.50,70.50) | 62.00(56.00,69.00) | -0.501 | 0.616 |
| Smoking status |  |  |  |  |
| Yes | 31(38.75%) | 53(36.05%) | -0.487 | 0.626 |
| No | 49(61.25%) | 94(63.95%) |  |  |
| Alcohol consumption |  |  |  |  |
| Yes | 25(31.25%) | 43(29.25%) | -0.907 | 0.364 |
| No | 55(68.75%) | 104(70.75%) |  |  |
| Severity status |  | | | |
| Non-severe | 39(48.75%) | 69(46.94%) | -1.220 | 0.223 |
| severe | 41(51.25%) | 78(53.06%) |  |  |
| ABI | 0.98(0.79,1.12) | 0.98(0.81,1.13) | -0.231 | 0.817 |
| eGFR(mL/min/1.73m^2^) | 77.30(50.20,101.35) | 73.40(48.13,99.00) | -1.448 | 0.148 |
| Duration of  diabetes (years) | 7.0(3.0,11.0) | 7.0(3.00,12.0) | -0.257 | 0.797 |
| HbA1c (%) | 8.10(6.50,10.10) | 8.40(7.20,10.90) | -1.674 | 0.094 |
| WBC(×10⁹/L) | 10.24(7.61,12.82) | 10.04(7.45,13.78) | -0.467 | 0.641 |
| CRP (mg/L) | 59.90(13.72,119.93) | 63.20(15.95,120.17) | -1.384 | 0.166 |
| Nutrition status |  |  |  |  |
| BMI | 22.69(20.56,25.23) | 22.71(20.50,24.83) | -1.035 | 0.301 |
| Hb(g/L) | 105.00(81.90,123.20) | 99.10(85.75,113.60) | -1.082 | 0.279 |
| Alb(g/L) | 33.47±6.54 | 31.54±5.80 | -1.833 | 0.067 |
| PA (mg/L) | 132.40(82.30,219.88) | 128.80(77.90,190.80) | -1.045 | 0.296 |
| Total cholesterol (mmol/L) | 3.68(3.32,4.37) | 3.86(3.21,4.61) | -0.383 | 0.702 |
| RBP (mg/L) | 32.60(19.20,47.10) | 30.52(20.35,43.87) | -0.744 | 0.457 |
| Serum Ca^2+^  (mmol/L) | 2.14(2.02,2.30) | 2.14(1.99,2.26) | -1.047 | 0.295 |
| 25(OH)D  (nmol/L) | 49.82 (31.24, 67.55) | 51.45 (32.41, 68.92) | -0.403 | 0.687 |
| Lym (×10⁹/L) | 1.64(1.22,2.24) | 1.59(1.14,2.08) | -1.132 | 0.257 |
| CD4/CD8 ratio | 1.52 (1.15, 2.20) | 1.56 (1.19, 2.26) | -0.225 | 0.822 |
| NLR | 4.38(2.72,6.44) | 4.48(2.48,9.60) | -0.830 | 0.407 |
| ANLR | 7.38(4.57,14.68) | 6.58(2.90,13.17) | -1.387 | 0.166 |
| CONUT | 3.00(0.00,5.00) | 3.00(1.00,6.00) | -1.574 | 0.116 |
| PNI | 40.25±11.58 | 39.40±8.03 | -1.275 | 0.202 |

Abbreviations: Non-severe DFU (Wagner 2/3), Severe DFU (Wagner 4/5); ABI, ankle–brachial index; eGFR, estimated glomerular filtration rate(CKD-EPI); BMI, body mass index; HbA1c, glycated hemoglobin; WBC, white blood cell count; Hb, hemoglobin; Alb, albumin; PA, prealbumin; CRP, C-reactive protein; RBP, retinol-binding protein; 25(OH)D, 25-hydroxyvitamin D; Lym, Lymphocyte count; NLR, neutrophil to lymphocyte ratio; ANLR, albumin/neutrophil to lymphocyte ratio; CONUT, controlling nutritional status; PNI, prognostic nutritional index.

**Supplementary Table 6. Perioperative changes in PNI and ANLR among patients undergoing TTT.**

| **Variable** | **Pre-TTT** | **Post-TTT** | **t/z** | **P value** |
| --- | --- | --- | --- | --- |
| ANLR | 7.35(3.38,13.81) | 13.57(8.67,20.25) | 5.876 | <0.001 |
| PNI | 39.54±7.50 | 45.69±7.01 | 9.579 | <0.001 |

Compared with preoperative levels, PNI and ANLR both exhibited significant positive changes 1 month postoperatively (P < 0.001). PNI and ANLR showed significant changes at 1 month after TTT compared with baseline values

| **Variable** | **Coefficient** | **Wald** | **P-value** | **HR** | **95%CI low** | **95%CI up** |
| --- | --- | --- | --- | --- | --- | --- |
| Sex | -0.216 | 1.116 | 0.291 | 0.806 | 0.539 | 1.203 |
| Age | -0.026 | 6.648 | 0.010 | 0.974 | 0.955 | 0.994 |
| Smoking status | 0.036 | 0.039 | 0.843 | 1.037 | 0.725 | 1.483 |
| Alcohol consumption | 0.105 | 0.303 | 0.582 | 1.110 | 0.765 | 1.611 |
| DFU severity | -0.414 | 5.736 | 0.017 | 0.661 | 0.471 | 0.928 |
| ABI | 0.349 | 0.706 | 0.401 | 1.418 | 0.628 | 3.623 |
| eGFR | 0.000 | 0.019 | 0.890 | 1.000 | 0.995 | 1.006 |
| Duration of diabetes | -0.016 | 1.626 | 0.202 | 0.984 | 0.961 | 1.008 |
| HbA1c | 0.020 | 0.357 | 0.550 | 1.020 | 0.956 | 1.088 |
| WBC | 0.003 | 0.055 | 0.815 | 1.003 | 0.978 | 1.029 |
| CRP | 0.002 | 1.997 | 0.158 | 1.002 | 0.999 | 1.004 |
| Nutrition status |  |  |  |  |  |  |
| BMI | 0.000 | 0.143 | 0.705 | 1.000 | 0.999 | 1.001 |
| Hb | 0.000 | 0.000 | 0.998 | 1.000 | 0.992 | 1.008 |
| Alb | 0.006 | 0.183 | 0.669 | 1.006 | 0.978 | 1.036 |
| PA | 0.001 | 1.464 | 0.226 | 1.001 | 0.999 | 1.004 |
| Total cholesterol | -0.045 | 0.316 | 0.574 | 0.956 | 0.818 | 1.118 |
| RBP | 0.001 | 0.101 | 0.751 | 1.001 | 0.993 | 1.009 |
| Serum Ca^2+^ | 0.596 | 1.589 | 0.207 | 1.815 | 0.718 | 4.583 |
| 25(OH)D | 0.577 | 1.474 | 0.233 | 1.782 | 0.704 | 4.051 |
| Lym | 0.082 | 0.416 | 0.519 | 1.085 | 0.846 | 1.391 |
| CD4/CD8 ratio | -0.049 | 0.156 | 0.693 | 0.952 | 0.784 | 1.213 |
| NLR | 0.001 | 0.005 | 0.946 | 1.001 | 0.974 | 1.026 |
| ANLR | 0.000 | 0.000 | 0.998 | 1.000 | 0.981 | 1.020 |
| CONUT | -0.004 | 0.022 | 0.882 | 0.996 | 0.939 | 1.055 |
| PNI | 0.009 | 0.580 | 0.446 | 1.009 | 0.986 | 1.032 |
| ΔANLR | 0.012 | 1.821 | 0.177 | 1.012 | 0.995 | 1.030 |
| ΔPNI | 0.027 | 6.439 | 0.011 | 1.027 | 1.006 | 1.048 |

**Supplementary Table 7. Univariable Cox proportional hazards analysis of factors associated with wound healing time after TTT.**

Abbreviations: Non-severe DFU (Wagner 2/3), Severe DFU (Wagner 4/5); ABI, ankle–brachial index; eGFR, estimated glomerular filtration rate (CKD-EPI); BMI, body mass index; HbA1c, glycated hemoglobin; WBC, White blood cell; Hb, hemoglobin; Alb, Albumin; PA, Prealbumin; CRP, C-reactive protein; RBP, retinol-binding protein; 25(OH)D, 25-hydroxyvitamin D; Lym, Lymphocyte count; NLR, neutrophil to lymphocyte ratio; ANLR, albumin/neutrophil to lymphocyte ratio; CONUT, controlling nutritional status; PNI, prognostic nutritional index; ΔANLR was calculated as postoperative ANLR minus preoperative ANLR; ΔPNI was calculated as postoperative PNI minus preoperative PNI.

**Supplementary Table 8. Assessment of the proportional hazards assumption for Cox regression models of wound healing time**

| **Model** | **Variable** | **χ²** | **df** | **P value** |
| --- | --- | --- | --- | --- |
| Final Cox model | severity | 3.976 | 1 | 0.046 |
| Final Cox model | ΔPNI | 0.631 | 1 | 0.427 |
| Final Cox model | Global | 5.128 | 2 | 0.077 |
| Stratified Cox model | ΔPNI | 1.39 | 1 | 0.24 |
| Stratified Cox model | Global | 1.39 | 1 | 0.24 |

DFU severity showed a slight violation of the proportional hazards assumption in the final Cox model; therefore, a stratified Cox model was constructed using DFU severity as the stratification factor. The stratified model satisfied the proportional hazards assumption.

**Supplementary Figure 2.** **Assessment of the proportional hazards assumption and validation of the stratified Cox model.**

**A.** Schoenfeld residual plot for DFU severity showing evidence of deviation from proportional hazards assumption

**B.** Schoenfeld residual plot for ΔPNI in the original Cox model.

**C.** Schoenfeld residual plot for ΔPNI after stratification by DFU severity, showing an acceptable proportional hazards pattern.

**Supplementary Table 9. Sensitivity analyses of the association between ΔPNI and subsequent wound healing after additional adjustment for perioperative albumin supplementation and 1-month inflammatory markers.**

| **Model** | **Adjustment** | **ΔPNI HR (95% CI)** | **P value** |
| --- | --- | --- | --- |
| Model 1 | Fully adjusted baseline model | 1.037 (1.009–1.065) | 0.009 |
| Model 2 | + Albumin supplementation (yes/no) | 1.035 (1.007–1.064) | 0.014 |
| Model 3 | + Cumulative albumin dose (per 10-g increase) | 1.036 (1.007–1.066) | 0.014 |
| Model 4 | + WBC and CRP at 1 month | 1.031 (1.001–1.061) | 0.039 |

**Model 1:** The fully adjusted baseline model included age, sex, smoking status, alcohol consumption, diabetes duration, HbA1c, ABI, BMI, eGFR, baseline CRP, and PA, with DFU severity as a stratification factor.

**Model 2**: Further adjusted for perioperative albumin supplementation (yes/no).

**Model 3:** Further adjusted for cumulative albumin dose (per 10-g increase) instead of supplementation status. Patients without albumin supplementation were assigned a cumulative albumin dose of 0 g.

**Model 4:** Further adjusted for WBC and CRP measured at 1 month after TTT.
